# Regulation of genetically engineered (GE) mosquitoes as a public health tool: a public health ethics analysis

**DOI:** 10.1186/s12992-021-00760-x

**Published:** 2022-02-21

**Authors:** Zahra Meghani

**Affiliations:** Philosophy Department, University of Rhode Island, Kingston, RI 02881 USA

**Keywords:** Mosquito-borne diseases, Genetically engineered mosquitoes, Risk assessments, Regulatory agencies, Global health governance, Social determinants of mosquito-borne diseases, Bill and Melinda Gates Foundation, Global Health Investment Fund, Neoliberal philanthropy

## Abstract

**Background:**

In recent years, genetically engineered (GE) mosquitoes have been proposed as a public health measure against the high incidence of mosquito-borne diseases among the poor in regions of the global South. While uncertainties as well as risks for humans and ecosystems are entailed by the open-release of GE mosquitoes, a powerful global health governance non-state organization is funding the development of and advocating the use of those bio-technologies as public health tools.

In August 2016, the US Food and Drug Agency (FDA) approved the uncaged field trial of a GE *Aedes aegypti* mosquito in Key Haven, Florida. The FDA’s decision was based on its assessment of the risks of the proposed experimental public health research project. The FDA is considered a global regulatory standard setter. So, its approval of the uncaged field trial could be used by proponents of GE mosquitoes to urge countries in the global South to permit the use of those bio-technologies.

**Method:**

From a public health ethics perspective, this paper evaluates the FDA’s 2016 risk assessment of the proposed uncaged field trial of the GE mosquito to determine whether it qualified as a *realistic* risk evaluation.

**Results:**

The FDA’s risk assessment of the proposed uncaged field trial did not proximate the conditions under which the GE mosquitoes would be used in regions of the global South where there is a high prevalence of mosquito-borne diseases.

**Conclusion:**

Given that health and disease have political-economic determinants, whether a risk assessment of a product is realistic or not particularly matters with respect to interventions meant for public health problems that disproportionately impact socio-economically marginalized populations. If ineffective public health interventions are adopted based on risk evaluations that do *not* closely mirror the conditions under which those products would actually be used, there could be public health and ethical costs for those populations.

## Background

In recent years, different types of genetically engineered (GE) mosquitoes have been proposed as public health measures against the high incidence of mosquito-borne diseases in regions of the global South. GE mosquitoes are intended to either change features of or suppress the population of their wildtype counterpart so that they cannot act as disease vectors. However, uncertainties as well as risks for humans and ecosystems are entailed by the open-release and even uncaged field trials of the GE mosquitoes (see, for instance, [1–3]). This article provides a public health ethics analysis of the efforts of the US Food and Drug Administration (FDA) to evaluate the risks of a proposed (uncaged) field trial in Key Haven, Florida of the OX513A GE *Aedes aegypti* mosquito.^1^

Specifically, this paper evaluates the FDA’s 2016 Environmental (Risk) Assessment [41] of the proposed open release experimental project to determine whether it qualifies as a realistic risk assessment. The criterion of realistic risk assessment differentiates between risk evaluations that do and those that do not offer relevant information about how a proposed public health tool will perform under real world conditions. A risk evaluation of a potential public health measure that does not mirror the actual conditions under which the product will be used may have questionable epistemic and public health value, and thereby, ethical worth. This paper proposes that the criterion of realistic risk assessment should be used to evaluate the risk assessments of uncaged field trials of GE mosquitoes conducted by any regulatory agency.

The case is made that the FDA’s Environmental (Risk) Assessment [41] of the proposed uncaged field trial of the OX513A GE mosquito does not meet the realistic risk assessment criterion. The field trial site is not an area where *Aedes aegypti*-borne diseases are endemic. So, the uncaged experimental research project would not provide information about how the OX513A GE *Aedes aegypti* mosquito would function in regions where there is a high prevalence of those diseases.

By evaluating the FDA’s Environmental (Risk) Assessment of the proposed uncaged field trial of the OX513A GE mosquito in Key Haven Florida, this paper means to ask the larger question *why* the sponsors of the bio-technology have sought to conduct uncaged field trials of their patented bio-products in the US. The US does not have a high incidence of mosquito-borne diseases. During 2004–2016, four US states had the highest reported incidence of mosquito-borne diseases; California had 9254 cases, New York had 7167 cases, Texas had 6649 cases, and Florida had 3822 cases [4]. To state the obvious, in the US there is not a significant incidence of mosquito-borne diseases relative to regions of the global South where mosquito-borne diseases are endemic.

This paper also analyzes the episodic outbreaks of dengue, an *Aedes aegypti*-transmitted infection, in Puerto Rico. During 2004–2016, the US territory had 80,534 reported cases of mosquito-borne diseases [4]. It is an instance of the type of real-world conditions under which there is a (relatively) high incidence of *Aedes aegypti*-borne infections *and* it serves as a telling comparison to the proposed field trial site of Key Haven, Florida. In Puerto Rico, economic and political factors play an important role in determining the incidence of that *Aedes aegypti*-transmitted infections. So, the question must be asked whether there is ethical warrant for efforts to use patented high-tech ‘solutions’ that carry considerable uncertainty and risks for humans and ecosystems to ‘solve’ public health problems that disproportionately affect economically and politically marginalized populations in regions of the global South.

Additionally, this paper argues that in the interest of conducting a realistic risk assessment, the FDA should have evaluated the possible impact of the OX513A GE *Aedes aegypti* mosquitoes establishing colonies in areas with marginalized populations who have limited or no access to health care.^2^ The World Health Organization (WHO) in part classifies mosquito-borne diseases as neglected diseases because the populations disproportionately affected by them tend to be politically and economically marginalized [5]. Undocumented farmworkers are discussed as an example of a marginalized population who have limited access to health care in the US and who could be affected by the uncaged release of GE mosquitoes. It is argued that the FDA ought to have considered the possible impact of the uncaged fields trial of the OX513A GE mosquitoes on them. That possibility needs to be examined because tetracycline is used in farms *and* if the OX513A GE *Aedes aegypti* mosquitoes are released near farms (or inadvertently transported to them) *and* if they find tetracycline in sufficient quantities there, the GE mosquitoes might be able to survive over multiple generations. That could have implications for undocumented farmworkers who have limited access to health care.

Next, the justification for this project is discussed. It is argued that this paper’s evaluation of the FDA’s risk assessment of the proposed uncaged field trial matters. The regulatory agency is considered a global standard setter. Neoliberal philanthropies that seek to ‘solve’ public health problems of the global South by means of patented high-tech innovations (such as GE mosquitoes) could use the FDA’s risk assessment to advance their ends. They could use it to urge other countries to permit uncaged field trials and use (of different kinds) of GE mosquitoes in their territories.^3^ If patented GE mosquitoes get uptake among global health governance actors as tools for reducing the incidence of mosquito-borne disease in regions of the global South, it could mean substantial profits for those who own the patents for them even though the bio-technologies present significant uncertainties and risks for humans and ecosystems.

Following the discussion of the ‘framing’ of patented GE mosquitoes as the solution to mosquito-borne diseases in the South, key features of the OX513A GE *Aedes aegypti* mosquito are outlined. It provides background information for this paper’s analysis of the FDA’s Environmental (Risk) Assessment of the uncaged field trail of the OX513A GE mosquito in Key Haven, Florida [41].

### The FDA’s risk assessment of GE mosquitoes and global health governance

The socio-political and economic conditions of individuals and groups affects their health and diseases status [6]. Whether the risk assessments of products conducted by regulatory agencies approximate actual conditions under which they will be used especially matters in the case of products that are intended to act as interventions for public health problems that disproportionately affect the poor, such as mosquito-borne diseases. This ethics analysis argues that if ineffective public health interventions are adopted on the basis of epistemically weak risk evaluations by regulatory agencies, there could be substantial public health and ethical costs for that population.

On the international stage, the US FDA is regarded as a regulatory standard setter, with various countries adopting its criteria and methods for evaluating pharmaceuticals [7]. So, the FDA’s decision to approve the uncaged field trial of a GE mosquito could have international significance. Proponents of the GE mosquito could use it to argue that other countries should permit uncaged field trials of different types of GE mosquitoes in their territories and employ the same parameters that the FDA used. This would be a particular concern with respect to low-income countries that do not have the well-funded and well-resourced regulatory agencies of high-income countries. Regulatory agencies of many countries are under pressure to harmonize their regulatory approaches. The strongest push to harmonize comes from the World Trade Organization, the Organization for Economic Co-operation and Development, and countries that espouse strong patent regimes (see, for instance, [8]).

The FDA’s approval of the OX513A GE *Aedes aegypti* mosquito as a public health tool could make it (and other kinds of patented GE mosquitoes) appealing to the Global Health Investment Fund and other such social finance enterprises that aim to ‘solve’ the public health problems of the global South poor by means of patented products. To understand the normative commitments of such ventures and their efforts to solve the public health problems of the global South poor, it is useful to examine their ideological underpinnings. They appear to be a mix of neoliberalism and neo-colonialism.

#### Neoliberal-philanthropic enterprises

Neoliberalism does not have a uniform form nor is it reproduced uniformly over time and contexts [9–11]. Rhetoric that disparages government intervention in the economy, lobbying for regulations that are pro-corporate interests, and valorization of the (mythical) bootstrapping, enterprising individual are some of the key practices of neoliberalism. But it is generally understood that not all of those features are present in each case of neoliberalism and no specific characteristic is common to all instances of neoliberalism [11].

However, some features do occur in many instantiations of the ideology. The tendency to construe the democratic state as a moral and practical failure is one such characteristic of neoliberalism. According to it, the democratic state is an unfair and inefficient allocator of social goods and services that discourages individuals from acting as self-sufficient, enterprising risk takers [12].

Some powerful non-state actors committed to capitalism or neoliberalism believe they can do better than the democratic state [13–16]. Thus, they have chosen to take on some of the roles of the state motivated by the conviction that they can be just, efficient, and effective [14, 16].

However, they are neither chosen by the populations whose lives they impact to take on those roles nor are they accountable to them. The lack of accountability does not seem to be viewed as an ethical or political problem by proponents of neoliberalism. So, while the ideology evinces commitment to the principle of moral equality of all persons, its norms, policies, and practices are inconsistent with it. The failure to recognize this inconsistency is an epistemic problem with neoliberalism that has ethical and political dimensions.

The rise of neoliberalism has also given birth to neoliberal-philanthropic schemes.^4^ Those enterprises aim to assume some of the functions of the state by providing for the needs of the poorer segments of the polity. They are based on neoliberal business principles and their proponents appear to see no ethical tension in serving the interests of their supporters (including funders) as they seek to attend to their philanthropic mission.^5^

Social (public health) finance enterprises can be classified as a sub-category of neoliberal philanthropic endeavors.^6^ While the financially and politically powerful organizations voluntarily take on some of the roles of the state, they do not consider themselves accountable to the populations whose problems they seek to solve nor are they chosen by them to take on that responsibility [17]. In general, social finance enterprises are predicated on the assumption that charitable giving should ‘work’ for the investors in their organizations by generating profits for them [17].

Neoliberal-philanthropic public health initiatives that focus on the global South tend to see it from a neo-colonial perspective. Mirroring the policies and practices of colonial public health schemes, they retain for themselves key decision-making power, even as they seek to solve the public health problems of the South (see, for instance, [18–20]). They appear to regard state actors (including national public health agencies) of the global South as corrupt, inefficient, and ineffective, and themselves as virtuous, effective, and efficient benefactors of the global South poor. Thus, they tend to retain decision-making power for themselves [19]. However, it is an open question whether such enterprises can realize their ambition of solving the public health problems of the global South poor, such as the high incidence of mosquito-borne diseases. In part because that goal may be at odds with their ambition of producing profits for their investors or benefitting their funders and supporters [21, 22].^7^

The Global Health Investment Fund (GHIF) qualifies as a form of social(public health) finance enterprise. With the aim of generating profits for its investors, GHIF invests in patented interventions aimed at solving public health problems that disproportionately impact socio-economically marginalized populations in the global South ([23]: S311). In 2014, the Fund’s website stated that its goal was to help “bring about significant improvements in the treatment and prevention of disease, and in family planning, and the reduction of maternal and child mortality (in poorer countries), along with the prospect of a net financial return for investors” ([24], quoted in [23]: S311).

Mosquito-borne diseases that disproportionately affect young children of poor families in regions of the global South are within the scope of the GHIF’s mission. GE mosquitoes as a patented public health intervention could be of considerable interest to GHIF and its investors. For instance, the Oxitec GE mosquito was expected to cost the Brazilian city of Piracicaba (with a population of 391,449) an estimated US $1.1 million during a two-year period at the cost of US $10 per person in the target area (50% of the cost was supposed to financed by the city’s current mosquito control budget; Oxitec was expected to cover some part of the cost [25]). As the GE mosquito is a patented product, presumably it would have to be re-licensed every season [26]. Another study suggested that the price of the GE mosquito for use in an urban area with a population of 50,000 would be an estimated US $1.9 million in the first year and US $384,000 each following year [27]. So, if those numbers are anything to go by, presumably, the patented GE mosquitoes could bring substantial returns for their investors.

The initial eleven investors in the GHIF, included the Bill and Melinda Gates Foundation, JP Morgan Chase, the World Bank, Merck, Pfizer Foundation, and GlaxoSmithKline ([23]: S311). The possibility that patented GE mosquitoes could receive the GHIF’s endorsement and support should not be dismissed out of hand. The Gates Foundation, a key investor in the GHIF, has funded Target Malaria in the amount of US $75 million [28]. That initiative is using techniques of modern biotechnology to develop GE mosquitoes with intentionally altered heritable traits (albeit of a different kind than the OX513A GE *Aedes aegypti* mosquito that was proposed for the uncaged field trial in Key Haven, Florida). Target Malaria aims to use its GE mosquitoes to wipe out malaria in regions of sub-Saharan Africa [28]. A 2016 *MIT Technology Review* article notes that “[t] he Gates Foundation has said it no longer believes that malaria can be wiped out without a (GE mosquito with) gene drive.^8^ ‘You can’t walk around with a bed net on you all the time. That’s not going to eliminate malaria,’ says (Fil) Randazzo (deputy director of the Foundation). With a (GE mosquito with) gene drive, ‘human behavior change is not required’” [29].

The sentiments of key Gates Foundation personnel should be taken seriously. The Foundation is a part of the GHIF and it is a powerful global health governance actor. It plays a crucial role in deciding which health care interventions should be adopted and funded for the poor of low-income countries [13, 30, 31]. As the use of GE mosquitoes as a public health tool has the support of a key global health governance actor, and given the credence internationally afforded to the FDA as a regulatory authority, the agency’s risk assessment of the OX513A GE *Aedes aegypti* mosquito as a potential public health tool could have a wide-ranging impact. Therefore, this ethics analysis argues that regulatory agencies have an epistemic, public health, and ethical duty to adopt a policy of conducting realistic risk assessments of bio-technologies that are meant to serve as public health measures. To provide a context for this paper's analysis, next, key features of mosquito-borne diseases and the OX513A GE *Aedes aegypti* mosquito are briefly described.

#### The OX513A GE *Aedes aegypti* mosquito

Mosquito-borne diseases (in conjunction with other vector-borne diseases) take an enormous toll on the poor in subtropical and tropical regions. The vulnerability of those populations to vector-borne diseases is partly determined by a complex of socio-political- economic-ecological factors, including global warming and capital-led deforestation ([6, 32], vi [13, 33–35]). Histories and cultures, including the affected population’s stance on risks from mosquitoes, also play a role in determining their vulnerability (see, for instance, [36]).

However, some proponents of GE mosquitoes construe the disproportionate incidence of those diseases among the socio-economically marginalized as a primarily, if not wholly, biological phenomenon that must be solved by means of high-tech interventions (see, for instance, [37]). Their stance is in keeping with a long tradition of certain global health governance actors biologizing the high incidence of infectious diseases among the poor of the global South and advocating purely technical solutions for them [8, 13, 38].

Developed by Oxitec Ltd. (Oxford Insect Technologies), the OX513A GE *Aedes aegypti* mosquito is meant to reduce the population of its wild-type counterpart. The *Aedes aegypti* mosquito is the main vector of the yellow fever virus, dengue viruses, chikungunya virus, and Zika virus [39]. *Aedes aegypti* mosquitoes are found in tropical and subtropical regions and tend to establish their habitats in or near human dwellings. They have been inadvertently transported around the globe by humans and *Aedes aegypti* sub-species are associated with specific regions [40]. Female *Aedes aegypti* mosquitoes bite (human and non-human) animals and transmit organisms that may cause illness; male mosquitoes do not bite animals ([41], p.16).

The genetic modification of the Oxitec OX513A GE *Aedes aegypti* mosquitoes includes a heritable synthetic genetic sequence that makes them dependent on tetracycline ([41], p.22). It is expected that when male OX513A GE *Aedes aegypti* mosquitoes (that are released in the wild during field trials or open-releases) mate with their female wildtype counterpart, approximately 95% of the resulting progeny will inherit the tetracycline dependency trait such that they are not expected to survive to adulthood in environments that do not have sufficient amounts of that chemical ([42], p.13). The assumption is that with periodic releases of additional batches of male OX513A GE *Aedes aegypti* mosquitoes, which mate with their wildtype female counterpart, the population of the wildtype *Aedes aegypti* in the target environment will decrease over time because their progeny will not be viable in environments that do not have tetracycline [41]. That is expected to result in a decline in the incidence of dengue and other diseases transmitted by that strain of mosquito.

While Oxitec’s plan was to release only male OX513A GE *Aedes aegypti* mosquito during the uncaged field trial in Key Haven, it estimated that .2% of the released GE mosquitoes could be females as the sex sorting system used to differentiate between male and female GE mosquitoes had deficiencies ([42] pg.34). So female GE mosquitoes (with the ability to transmit organisms that could cause infections in humans) could be accidentally released. To get a sense of those percentages, it is useful to consider the actual number of OX513A GE mosquitoes that would have been released during the proposed uncaged Key Haven field trial. During the 22 months long experimental research project, Oxitec anticipated releasing in the trial site at least 14 million mosquitoes ([41], p.39). That means if the field trial had been conducted, then it was possible that over time approximately 28,704 female GE *Aedes aegypti* mosquitoes could have been inadvertently released. If they had found sufficient tetracycline in the environment, presumably, they and their GE progeny could have survived, established habitats, and the female GE mosquitoes and the female GE progeny of female or male GE mosquitoes could have acted as disease vectors.

Although the uncaged field trial of the OX513A GE *Aedes aegypti* mosquito in Key Haven, Florida was greenlighted by the FDA, it was *not* conducted because the local population objected [43]. However, this paper’s ethics analysis of the FDA’s risk assessment of the proposed field trial matters because, first, it identifies serious shortcomings with the agency’s risk evaluation, and second, it may have relevance for *other* proposed uncaged field trials of GE mosquitoes in various countries including the US. In 2020, two uncaged field trials in Florida and Texas of a *different* GE *Aedes aegypti* mosquito (OX5034)^9^ were approved by the US Environmental Protection Agency (EPA).^10^ An estimated 508,560,000 male OX5034 GE *Aedes aegypti* mosquito are to be released as part of uncaged field trials in Florida, and approximately 249,600,000 male OX5034 GE *Aedes aegypti* mosquito are to be released in Texas for an uncaged experimental field research project [44], p. 5. It is beyond the scope of this paper to address the question whether the EPA’s risk assessment qualified as a realistic risk assessment. However, as argued earlier, it is a criterion by which the EPA’s decision to authorize the uncaged field trials of the OX5034 GE mosquito should be evaluated.

## Method

This paper evaluates the FDA’s risk assessment of the OX513A GE mosquito to determine whether it qualifies as a *realistic* risk assessment of the proposed public health measure.^11^ The argument is made that regulatory agencies should have a policy of conducting realistic risk evaluations of bio-technologies intended to serve as public health tools.^12^

The concept of *realistic* risk assessment serves to distinguish between risk evaluations that do and those that do not provide relevant information about how a proposed public health measure will perform under real world conditions. A risk assessment of a proposed public health tool that does not mirror the actual conditions under which the product will be used may have questionable epistemic and public health value, and thereby, moral worth.

This paper’s analysis is also informed by the scholarship that examines the efforts of North-based actors to solve public health problems that disproportionately affect the poor of the South. The colonial and neoliberal underpinnings and shape of such public health schemes have been analyzed at length (see, for instance, [13, 18, 46–49).

Based on that analytic framework, this public health ethics analysis does four things. First, it identifies some of the marginalized populations that could be affected by the uncaged field trials or open-releases of GE mosquitoes. Second, it raises epistemic and ethical questions about risk assessment reports that frame patented, expensive high-tech technological products as easy solutions to complex public health problems, without acknowledging their socio-political dimensions. Third, it asks the question whether efforts to solve public health crises that disproportionately affect the poor should be influenced by concerns about generating profits for organizations that have invested in particular ‘solutions.’ Fourth, it brings to light the larger issue of whether non-state actors (with a neoliberal or neoliberal-neo-colonial worldview) should be making decisions about public health problems of populations to whom they are not accountable and who were not chosen by them to make those decisions for them.

This paper’s ethics analysis stands in contrast to Meghani and Kuzma’s [50] evaluation of the risk assessment of the proposed field trial of the OX513A GE *Aedes aegypti* mosquito. That paper examined the risk evaluation of the proposed field trial that was prepared by Oxitec, the developer of the OX513A GE mosquito. This paper does not evaluate Oxitec's *Draft Environmental (Risk) Assessment* [42], although it should be evaluated using the realistic risk assessment criterion introduced in this paper. Instead, the FDA’s risk assessment report of the proposed field trial of the OX513A GE *Aedes aegypti* mosquito is evaluated on ethical and epistemic grounds. Moreover, unlike Meghani and Kuzma [50], this ethics analysis argues that the location of the proposed field trial means that the FDA would not be able to generate a realistic assessment of the risks from the use of the GE insect as a public health tool in socio-economically marginalized regions with substantial prevalence of vector-borne diseases.

It should be noted that the FDA’s 2016 Environmental (Risk) Assessment does not appear to have drawn on the 2014 *Guidance Framework for Testing of Genetically Modified Mosquitoes* that has the imprimatur of the World Health Organization (WHO) and the Foundation of the National Institute of Health. (Henceforth the document is referred to as the WHO 2014 *Guidance.*) The WHO 2014 *Guidance* [52] is not in the list of references of the FDA’s 2016 *Environmental (Risk) Assessment* [41]. As a regulatory agency of a sovereign nation, the FDA is not obligated to use guidance documents issued by the WHO, the public health agency of the United Nations.

Sandra Schwindenhammer [51] provides a detailed critical evaluation of the involvement of Oxitec in shaping the WHO 2014 *Guidance* [52]. Her analysis in effect calls into question the epistemic and ethical independence of the WHO 2014 *Guidance* [52] given Oxitec’s substantial influence on it. In other words, she can be read as raising the question whether the document is biased towards the use of GE mosquitoes and the developers of the patented biotechnology.

It is also worth considering that the Core Working Group (that played a significant role in crafting the WHO 2014 *Guidance)* included persons who voluntarily disclosed their professional interest in GM mosquitoes ([52], p.131) and who were or had been affiliated in some capacity with the Gates Foundation.^13^ The Gates Foundation’s role in shaping the project that resulted in the WHO 2014 *Guidance* [52] has a long history. It can be traced back to at least 2009, when it provided funding support for the development of the guidelines for field trials of GM mosquitoes [53].

Oxitec and the Gates Foundation have also collaborated, with the former receiving funding from the latter (see, for instance, [54]). The significant involvement of Oxitec and the Gates Foundation in shaping the WHO 2014 *Guidance* [52] raises questions about a possible conflict of interest.

## Results

### A public health ethics evaluation of the FDA’s 2016 environmental (risk) assessment of the proposed uncaged field trial of the OX513A GE mosquito

This article evaluates the FDA’s 2016 Environmental (Risk) Assessment report (of the proposed uncaged field trial) of the OX513A GE *Aedes aegypti* mosquito. It aims to determine whether that report provides *relevant* data about the safety and efficacy of that GE mosquito as a public health measure in areas with high incidence of *Aedes aegypti*-borne diseases and where tetracycline may be present in the environment. The question about the presence of tetracycline in the environment matters because the ability of the OX513A GE *Aedes aegypti* mosquito to function as a public health tool depends on the lack of tetracycline in the environment [41]. If tetracycline is present in sufficient quantity, both female and male OX513A GE *Aedes aegypti* mosquito would survive, and presumably, the former could act as disease vectors as could any female progeny of female or male OX513A GE *Aedes aegypti* mosquitoes that inherited the tetracycline dependency trait.

The proposed goals of the uncaged field trial in Key Haven, Florida included the following ([41], pgs. 16, 91-97, 101)):

1. To evaluate whether the OX513A GE *Aedes aegypti* mosquitoes would mate successfully with the local wildtype *Aedes aegypti* mosquito in the field trial area and whether the progeny with the heritable tetracycline trait would survive.
2. To assess whether multiple releases over time of male OX513A GE mosquitoes would result in a decrease in the population of the wildtype mosquito population.
3. To determine if the proteins produced by OX513A GE *Aedes aegypti* mosquitoes were harmful to humans and other animals exposed to them ([41], pgs. 91-7).
4. To ascertain whether the GE *Aedes aegypti* mosquito could worsen the public health problem of dengue and other diseases transmitted by the *Aedes aegypti* strain of mosquitoes ([41], pg. 101).

Given that the goals of the uncaged field trial of the GE *Aedes aegypti* mosquito were to evaluate its safety for human health, and its ability to not worsen the incidence of *Aedes aegypti*- borne diseases, like dengue, under real world conditions (Goal 4), Key Haven was *not* the ‘right’ location for the experimental public health research project. It is a relatively affluent area with no reported cases of dengue or Zika. So, it would not provide data about whether the OX513A GE mosquito was effective in reducing *Aedes aegypti* mosquito transmitted infections or not worsening them.

It is worth noting that as justification for the proposed uncaged field trial in Key Haven, the FDA’s Environmental Assessment cited reported cases of dengue in *other* parts of Florida ([41], p.18). In 2009, there were 22 reported cases of the dengue virus, and in 2010, another 66 reported cases, in addition to other cases in Miami-Dade and Broward counties and an estimated 1000 people in the Florida Keys had been exposed to the dengue virus (approximately 5% of the population) ([41], p.18).

So, arguably, the decision to propose Key Haven as the site of the (uncaged) field trial of the GE *Aedes aegypti* mosquito is as inappropriate as the decision to propose an area where there is no fire (and virtually no risk of fire) as the site of the field test of a product that is supposed to prevent fires and act as a fire retardant. In both cases, arguably, the *location* of the field trial makes it unlikely that the project would yield *relevant* data about the safety or efficacy of the product under real world conditions (more on this below).

As mentioned earlier, in 2020, the US EPA authorized uncaged field trials of the OX5034 GE *Aedes aegypti* mosquitoes in Florida and Texas [45]. Whether it conducted a realistic risk assessment of those proposed uncaged field trials is an important question that lies beyond the scope of this project, which is limited to the FDA’s risk assessment of the proposed uncaged field trial of the OX513A GE *Aedes aegypti* mosquito. However, the EPA’s decision to authorize the uncaged field trial of the OX5034 GE mosquito should be evaluated to determine whether it met the realistic risk assessment criterion.

Next, the periodic outbreaks of dengue in Puerto Rico, a US territory, are discussed. It is an example of the sort of real-world conditions under which there is a high incidence of *Aedes aegypti*-borne infections *and* it serves as a telling contrast to the proposed field trial site of Key Haven, Florida. In Puerto Rico, economic and political factors play a role in determining the incidence of that *Aedes aegypti*-transmitted infections. So, it raises the question whether there is ethical warrant for efforts to use a high-tech ‘solution’ that presents considerable uncertainty and risks for humans and ecosystems.

## Discussion

### Dengue in Puerto Rico

The Dengue Branch of the US Centers for Disease Control and Prevention (CDC) is in Puerto Rico. Since 1963 there have been dengue epidemics in Puerto Rico [55, 89]. In non-outbreak years, approximately three to nine thousand suspected cases have been reported [55]. There have been five dengue epidemics or outbreaks since 1990 in the US Territory [55, 89] (Table 1).

**Table 1:** [55, 88]

Year	Reports of suspected dengue cases in Puerto Rico
1994	24,700
1998	17,000
2007	10,508
2010	26,766
2012-13	30,921

It is illuminating to contrast the incidence of mosquito-borne diseases in Puerto Rico, Florida, California, New York, and Texas (see Table 2). In 2016, the year of the Zika outbreak, Puerto Rico had 35,781 reported cases of mosquito-borne diseases (primarily Zika) [56].

**Table 2:** (CDC 2018) [4]

State	Reported cases of mosquito-borne diseases during 2004–2016 period
Puerto Rico	80,534
California	9254
New York	7167
Texas	6649
Florida	3822

The high incidence of dengue and Zika in Puerto Rico is not a matter of chance. Rather it is a telling combination of socio-political-economic-climatic conditions under which there tends to be significant prevalence of mosquito-borne diseases. In the US, Puerto Rico has the highest poverty level relative to that of US states and other territories, with approximately 45% of its residents living at or below the federal poverty level ([57], p.1). The median annual household income in Puerto Rico is approximately two-thirds less than the US median income ([57], p.1). The contrast between Puerto Rico and Florida, including Key Haven, reflects that disparity with respect to the median income (Table 3). Although this is not a case of apples to apples comparison, it is telling to an extent.

**Table 3:** (US Census Bureau [58])

State/city	Median household income (in 2017 dollars) during the 2013–2017 period
Puerto Rico	$ 19,775
Florida	$ 50,883
Key Haven (Coconut Creek city), Florida	$ 56,556

Presumably, the poverty in Puerto Rico is attributable in no small part to its political marginalization within the US political system. It is an unincorporated territory of the US such that while persons born in Puerto Rico are American citizens, they cannot vote in presidential elections (they are permitted to vote in the primary elections of political parties). Moreover, the territory cannot vote in Congress, thus, its political agency and thereby ability to secure federal resources is severely curtailed, with significant consequences for its population’s socio-economic and health status [59].

Puerto Rico’s climate in conjunction with its political marginalization has translated into a recurring pattern of outbreaks of mosquito-borne diseases. According to a 2016 Natural Resources Committee report on Puerto Rico, “many public schools, public housing, and even hospitals do not have air conditioning or proper window screens, leaving buildings open to mosquitos” [90], p.5. Moreover, approximately half of Puerto Rican homes rely on septic tanks, “thousands of which were never properly sealed to prevent mosquitos from breeding” [90], p.5 (see also [60]). The report also noted that there were hundreds of thousands of unused tires in Puerto Rico ([90], p.5). The tires collect water, serving as preferred breeding ground of mosquitoes. (Presumably, the local government did not have the resources to safely dispose of the tires.)

Matysiak & Roess [61] have clarified the complex relationship between climatic, ecologic, and socio-political factors shaping the periodic emergence of dengue and the incidence of Zika in Puerto Rico. They have argued that aside from mosquito population and infection surveillance, the government should provide all residents of the territory with piped water, regular garbage disposal service (so that containers, including unused tires, do not serve as breeding grounds for mosquitoes), and “convert homes from open sewage to sewer use” ([61], p.12). The US Centers for Disease Control has noted that “it was recently found that *Ae.* (i.e., *Aedes*) *aegypti* (mosquito) is able to undergo immature development in broken or open septic tanks in Puerto Rico, resulting in the production of hundreds or thousands of *Ae. aegypti* (mosquito) adults per day” [62].

So, in contrast to US states, the US territory of Puerto Rico has an urgent need for *socio-political and economic interventions* to reduce the incidence of dengue and Zika. This analysis of the socio-political and economic components of the outbreaks of *Aedes aegypti-borne* diseases in Puerto Rico raises questions about the ethical warrant of proposals to use GE mosquitoes to reduce the incidence of those infections in socio-economically marginalized regions.

### Criteria for a realistic risk assessment of the OX513A GE mosquito

For the sake of a realistic risk assessment of the uncaged field trial of the OX513 GE mosquito as a public health tool to reduce the high incidence of *Aedes aegypti* borne-diseases, the experimental project would have to be proposed for a location that met at least two conditions. First, the field trial area would have to approximate circumstances under which *Aedes aegypti* transmitted diseases are endemic. In other words, it would have to be sited at a location where a number of persons might have *Aedes aegypti* transmitted viruses. That would allow for a meaningful assessment of the possibility of (inadvertently released) female GE *Aedes aegypti* mosquitoes or female GE progeny (of female or male GE mosquitoes) acquiring viruses when they feed on infected persons and transmitting them to others.

A risk assessment that reckoned with that possibility would have to include a detailed plan to track infections in humans that were attributable to (any inadvertently released) female GE *Aedes aegypti* mosquitoes or any female GE progeny of male or female GE mosquitoes. It would also track the interaction between the GE mosquito transmitted infections and other infections common in that population. This is an ethical requirement and good research practice. In fact, it is standard practice for the FDA to require report of adverse events during trials of pharmaceutical and medical devices products. So, it stands to reason that the same standard should be applied to these bio-technologies that are meant to act as a public health tool against mosquito-borne diseases. Moreover, any regulatory agency that authorizes the uncaged field trials or use of GE mosquitoes should require reports of adverse events attributable to the bio-products they regulate.

The other criterion for a realistic risk assessment of the OX513A GE mosquito would have to contend with the very real possibility of tetracycline in the environment. In the US and a number of other countries, tetracycline, an antibiotic, is overused for human therapeutic purposes. In 2018, in the US, it also constituted the largest volume of medically important antimicrobials sold or distributed for livestock (3,974,179 kg) and, during 2017–18, the chemical’s use increased by 12% ([63], p.3).

One of the ways in which tetracycline enters the environment is as waste from pharmaceutical manufacturing. Waste from pharmaceutical manufacturing facilities “contain 10–1000 times higher pharmaceuticals concentrations than other wastewaters … ” [64].^14^ In fact, various kinds of antibiotics have been found in untreated wastewater in many parts of the world (see, for instance, the review article by Daghrir and Drogui [65] on the ubiquity and persistence of tetracycline in aquatic and terrestrial environments; also see [66].

Given the widespread inappropriate use of antibiotics in animal feed for prophylactic purposes [67] *and* given the use of tetracycline on fruit farms, it is possible that there might be tetracycline in the untreated wastewater of farms. The presence of tetracycline could allow the tetracycline dependent OX513A GE mosquitoes to survive and establish colonies (see, for instance, [68]). So, a realistic risk assessment of the OX513A GE *Aedes aegypti* mosquito would factor in the possibility of tetracycline in the environment.

### Risks to marginalized populations: a public health and ethical issue

It is unlikely that the uncaged field trial in Key Haven would provide information about whether the modified mosquitoes could establish colonies under real world circumstances where tetracycline use is common and considerable. The FDA’s Environmental Assessment of the proposed uncaged field trial in Key Haven stated that in so many words: [A]quaculture facilities, farms, hospitals, or municipal sewage facilities are the only sources that theoretically could introduce into the environment sufficiently high levels of tetracycline to allow survival of OX513A (GE mosquito) progeny in the environment … (but there are) … no farms, including aquaculture facilities or citrus groves, or hospitals/medical centers in the proposed trial site” ([41], p.112).

In the interest of conducting a realistic risk assessment, the FDA ought to have assessed the possible impact of the OX513A GE *Aedes aegypti* mosquitoes establishing colonies in regions with marginalized populations. As mentioned earlier, the World Health Organization in part classifies mosquito-borne diseases as neglected diseases because the populations disproportionately affected by them in tropical and sub-tropical regions tend to be politically and economically marginalized (see, [5]). Farmworkers who have limited access to health care qualify as an example of a marginalized population in the US that could be affected by the uncaged release of GE mosquitoes. It is argued that the FDA ought to have considered the possible impact of the uncaged fields trials of the OX513A GE mosquitoes on them.^15^

If the OX513A GE *Aedes aegypti* mosquitoes are released near farms (or inadvertently transported to them) and if they find tetracycline in sufficient quantities there, they might be able to survive over multiple generations. In the interest of conducting a realistic risk assessment, the FDA should have considered what that would mean for farmworkers who have limited access to health care.

That worry, presumably, may have underlain the May 13, 2016 comment submitted to the FDA by Friends of Earth [69]. The civil society organization submitted a detailed letter in response to the FDA’s request for public comments on the February 2016 *Draft Environmental Assessment* report for the OX513A GE *Aedes aegypti* mosquito (the draft risk assessment was prepared by Oxitec [42]). Among other things, the civil society organization noted that the EPA was considering permitting increased use of tetracycline in Florida as a pesticide in citrus farms and if that request was approved by the EPA and if the OX513A GE mosquitoes were (inadvertently) transported to those farms, the GE mosquitoes could establish habitats in those locations [69]. Tetracycline is commonly used in agriculture and that, earlier in 2016, on behalf of citrus growers, the Florida Department of Agriculture and Consumer Services had requested the EPA’s permission to increase the use of tetracycline in citrus farms to control the bacteria that causes Citrus Greening disease (Docket No. EPA-HQ-OPP-2015-0849-0001). The civil society organization expressed the worry that if the EPA permitted increased use of tetracycline in agriculture in Florida, then hundreds of thousands of pounds of the chemical would be spread on citrus farmlands, obviating “the lethal trait in the GE mosquitoes and their offspring could survive and continue to breed” [69].

In 2016, there were no citrus farms in Key Haven (the proposed field trial site). However, according to the United States Department of Agriculture's National Agricultural Statistics Service, in 2015–16, a total of 737,800 acres in the US were devoted to citrus crops (737,800), with 435,300 acres (58.9%) in Florida ([91], p.8).

Citrus crop harvesting is labor intensive. As each fruit must be picked by hand, citrus farms employ significant numbers of farmworkers. If the OX513A GE *Aedes aegypti* mosquitoes were inadvertently transported to or areas near those farms and established habitats there, farmworkers could be exposed to them. As mentioned earlier, given that tetracycline is also used in the US on livestock farms for prophylactic and therapeutic purposes [63], the FDA’s risk assessment should have contended with the possibility of the OX513A GE *Aedes aegypti* mosquito establishing in those areas and its significance for farmworkers and their families, a socio-economically marginalized population.

Farmworkers generally live close to the lands on which they work. In a 2017 report, the National Farm Worker Ministry has noted that substandard housing is a reality and worry for farmworkers [70]. Low wages for farmworkers and inadequate and fragmented social protection programs mean that many farmworkers have to live in crowded, unsanitary housing that “often lack basic utilities, (and are in) … isolated areas far away from important services like health clinics ...” [70].

Farm owners in the US rely on undocumented workers to do labor intensive work that citizens or permanent residents are unwilling to take on, such as harvesting fruits and vegetables, and milking cows. The US Department of Agriculture estimated that during the 2012–14 period, approximately 47% of crop farmworkers were unauthorized persons [71]. 16Those farmworkers and their children are more likely than documented farmworkers to live in substandard housing because of their irregular immigration status [70]. The US limits undocumented persons’ access to publicly funded health care because of their irregular immigration status. If GE mosquitoes establish habitats near farm fields where tetracycline is used, farmworkers and their families (including those who are undocumented) could be exposed to them. Their limited access to health care could compound the risk to them.

The FDA’s Environmental Assessment did not consider the risk to farm laborers (including those who are undocumented and their families which may include young children) from the establishment of habitats of the OX513A GE *Aedes aegypti* mosquito in citrus farms and other kinds of farms where tetracycline is used in significant amounts and where climatic conditions are suitable for that strain of mosquitoes. That omission is ethically and politically significant and it results in a risk assessment that is epistemically weak. Specifically, it does not provide realistic data about the safety and efficacy of the GE mosquito in regions with marginalized populations at risk of mosquito-borne diseases and where tetracycline may be present in sufficient quantities in the environment.

The FDA may have been trying to minimize the risk from the uncaged field trial of the OX513A GE *Aedes aegypti* mosquito by authorizing it for Key Haven, Florida. It is a location where *Aedes aegypti* mosquito-borne diseases are non-existent and there is low probability of tetracycline in the environment so that even if female GE mosquitoes were inadvertently released during the experimental field research project, it was unlikely that they would survive and act as diseases vectors, and GE mosquitoes would establish colonies in or near that area. However, the authorization of Key Haven as the site of proposed uncaged field trial is a double-edged sword because it means that the risk assessment does *not* provide relevant data about the safety and efficacy of GE mosquitoes in real world conditions. From an ethics perspective, it does not qualify as a realistic risk assessment of the proposed public health tool.

Whether the FDA’s risk assessment is realistic or not matters because the regulatory agency is an international regulatory standard setter, and the Gates Foundation, a key global health governance actor, has espoused GE mosquitoes as the solution to the high incidence of mosquito-borne diseases in regions of the global South. The agency’s risk assessment of the GE mosquito could be (mis)read as indicating that the bio-technology can be used as a safe and efficacious public health tool against mosquito-borne diseases, including in regions of low and lower middle-income countries where mosquito-borne diseases are a significant public health problem and where tetracycline may be in the environment. That could motivate GHIF and other such social (public health) finance enterprises to push for the use of GE mosquitoes in regions of the global South where mosquito-borne diseases disproportionately affect the poor. To prevent such (mis)readings, there is an epistemic, public health, and ethical imperative on regulatory agencies to adopt a policy of conducting realistic risk assessments of proposed (uncaged) field trials of GE mosquitoes.

## Conclusion

This paper evaluated the FDA’s 2016 Environmental Assessment report of a proposed uncaged field trial of the OX513A GE mosquito. It was argued that the risk assessment report prepared by the regulatory agency did not qualify as a realistic assessment *because* the uncaged field trial was proposed for an area that did not proximate the real-world conditions under which the OX513 GE *Aedes aegypti* mosquito would be used to try to reduce *Aedes aegypti* transmitted infections. The FDA may have authorized the experimental bio-technology research project at such a site so as to substantially reduce the risks from the field trial. However, it also meant that the risk assessment of such a field trial would not provide an accurate evaluation of the risks from the use of the OX513A GE *Aedes aegypti* mosquito in regions where there is a high incidence of *Aedes aegypti* transmitted infections in the population.

This ethics analysis identified some of the marginalized populations that could be affected by the uncaged field trials of GE mosquitoes as well as open-release of those bio-technologies. It also raised questions about the epistemic and ethical value of risk assessment reports that present patented, expensive high-tech bio-technologies as simple solutions to complex multi-dimensional public health problems that disproportionately affect the global South poor. In addition, it asked the question whether non-state actors (with a neoliberal or neoliberal-neo-colonial orientation) should be making decisions about public health problems of populations to whom they are not accountable and who were not chosen by them to make those decisions for them.

By evaluating the FDA’s risk assessment of the proposed uncaged field trial in Key Haven, Florida *and* by identifying the low-incidence of mosquito-borne diseases in the states of the US, this paper raised the question why the sponsors of the bio-technology seek to conduct uncaged field trials in the US. It was argued that proponents of the biotechnologies may be interested in using the fact that a US regulatory agency authorized the uncaged field trials of GE mosquitoes to urge other countries to permit uncaged field trials and commercial use of the patented bio-technologies.

Another key aim of this paper has been to argue that given that health and disease have political-economic determinants, whether a risk assessment of a product is realistic or not particularly matters with respect to interventions meant for public health problems that primarily impact socio-economically marginalized populations. If ineffective interventions are adopted based on risk evaluations that do *not* approximate conditions under which the product would actually be used, there could be public health and ethical costs for those populations.

## Data Availability

Not applicable.

## Footnotes

1 In 2020, the US Environmental Protection Agency authorized uncaged field trials in Florida and Texas of a different GE mosquito, i.e., the OX5034 GE Aedes aegypti mosquito [45]. However, evaluating that agency’s risk assessment of those experimental projects lies beyond the scope of this paper [45].

2 As mentioned above, this criterion should be used to evaluate the risk assessment of uncaged field trials of GE mosquitoes conducted by any regulatory agency.

3 Arguably, those neoliberal-philanthropies could also use the fact that the US Environmental Protection Agency (EPA) authorized uncaged field trials of the OX5034 GE *Aedes aegypti* mosquito (in Florida and Texas) to ‘urge’ other countries to permit uncaged field trials and commercial use of GE mosquitoes. However, it is not obvious if the environmental regulatory agency’s risk assessment will carry significant weight internationally given that in recent years the EPA has come under the spotlight for serving Monsanto’s interests with respect to its herbicide Roundup that is intended for use with genetically modified seed that are tolerant of the chemical (see, for instance, [72], and [73]).

4 Philanthropic ventures, including those that attempted to ‘solve’ public health problems of the global South, predate neoliberalism (see Birn [18] on the history of the Rockefeller Foundation as a colonialist capitalist-philanthropical enterprise, also see Packard [46] for a detailed history and analysis of colonial public health ventures).

5 Based on Birn’s [18] and McGoey’s [15] analysis of the Gates Foundation, the private philanthropic organization qualifies as a neoliberal-philanthropic venture.

6 The term ‘social’ in ‘social finance enterprises’ refers to social goods or programs needed by socio-economically marginalized populations [17].

7 Also see Packard [46] on the epistemic problems with the analysis of public health problems of the global South that such enterprises draw on.

8 The term ‘(synthetic) gene drive’ generally refers to genetic modification (by means of techniques of modern biotechnology) that introduce into organisms intentionally altered heritable traits that are passed on to the progeny of those organisms at a rate greater than the one described by Mendel’s law of inheritance [3, 74–77]. The aim of this type of genetic modification is to affect population-level change in species by releasing such modified organisms in the wild. When the modified organisms mate with their wildtype counterparts, their progeny inherit the intentionally altered heritable traits at a rate greater than the one described by Mendel’s law, and the rate they pass it on to their offspring is also biased. The OX513A GE *Aedes aegypti* mosquitoes pass on the intentionally altered heritable traits (that have been introduced into them) to their progeny at a rate greater than the one described by Mendel’s law of inheritance ([42]; [41], pgs. 17-18)). However, in the absence of tetracycline in the environment, the modified mosquitoes and their progeny that inherit the intentionally altered heritable traits are not expected to survive.

9 The OX5034 GE *Aedes aegypti* mosquito's tetracycline dependency trait is sex linked such that while it is carried by all GE progeny of the OX5034 GE *Aedes aegypti* mosquitoes, the trait is expressed only in the female OX5034 GE Aedes aegypti organisms [78]).

10 In 2017, Oxitec’s application for the field trial of the OX513A GE mosquito was transferred to the US EPA. The switch in jurisdiction of the OX513A GE *Aedes aegypti* mosquitoes from the FDA to the EPA occurred because it was decided by the US that regulatory jurisdiction depends on how applicants classify their products. If a bio-product is classified by an applicant as a bio-pesticide (rather than a means for preventing or reducing the incidence of mosquito-borne diseases in humans), it will be regulated by the EPA and not the FDA [79]. Oxitec later withdrew its application for the uncaged field trial of the OX513A GE *Aedes aegypti* mosquito that it had submitted to the EPA, and submitted instead to the environmental agency an application for the uncaged field trials of a different GE mosquito, the OX5034 GE mosquito. The application for the uncaged field trials of that mosquito in Florida and Texas was approved by the EPA [78].

11 This paper does not take up the risks or uncertainties with respect to other animals or ecosystems because it focuses on the public health risks and uncertainties. However, it must be acknowledged that given the entangled nature of species and ecosystems, the bracketing of those concerns is an imperfect heuristic strategy (see [80] for a critique of such an approach).

12 The US FDA is not the only regulatory agency to have conducted a risk assessment of uncaged field trials of the OX513A GE mosquito. In 2010–11, Oxitec conducted a release in Malaysia in an uninhabited forest region, with regulatory authorization. Oxitec also performed field trials in the Cayman Islands even though the British territory “had no regulations concerning the release of GM organisms into the wild” ([81], p.140). The Cayman Island and Malaysian trials were subject to serious criticism (see [82], p.728). In 2010–2011, a Brazilian study of the entomological effectiveness of the OX513A GE mosquito was conducted by Oxitec in conjunction with Brazilian researchers (for a critical analysis of that project, see [83]). In 2017, the Netherlands’ National Institute for Public Health and the Environment published a technical evaluation of a proposed release of OX513A *Aedes aegypti* mosquitoes on the island of Saba [84]. It did not evaluate the efficacy of the use of the OX513A GE mosquito or its socio-economic ramifications. Nor did it examine the question of “the desirability of using these mosquitoes” ([84], Synopsis, p.3). France has also considered the use of GE mosquitoes. In May 2017, the French High Council for Biotechnology issued a scientific opinion about the use of genetically modified mosquitoes for vector control (see [85]).

13 Some of the other persons who were also involved in crafting or reviewing the WHO 2014 *Guidance* [52] had direct or indirect connections to the Gates Foundation too.

14 A 2014 Caribbean Business article identified Puerto Rico as the “fifth largest area in the world for pharmaceutical manufacturing with more than 80 (manufacturing) plants …” [86]. It is not known whether tetracycline is manufactured in Puerto Rico, but the capacity of wastewater treatment facilities to treat effluent from pharmaceutical facilities has been a serious ongoing public health and environmental concern for the territory (see, for instance, [87]).

15 This section clarifies why the establishment of OX513A GE *Aedes aegypti* mosquito colonies is a concern with respect to farms. However, it does not mean that other areas where tetracycline might be in the environment (such as locations where wastewater from pharmaceutical facilities is discharged, aquaculture facilities, hospitals, or municipal sewage facilities) are not a cause of concern.

16 According to a 2010 Southern Poverty Law Center report, 6 out of 10 farmworkers were undocumented ([88], p.4).
